# Somatic Mosaicism in Blaschkolinear Inflammatory Disorders

**DOI:** 10.4172/2155-9554.1000356

**Published:** 2016-06-05

**Authors:** Nicholas Theodosakis, Lauren Levy, Shawn Cowper, Rossitza Lazova, Lisa Kugelman, Amanda Zubek, Keith Choate

**Affiliations:** 1 Department of Dermatology, Yale School of Medicine, New Haven, CT, USA; 2 Department of Pathology, Yale School of Medicine, New Haven, CT, USA; 3 Department of Genetics, Yale School of Medicine, New Haven, CT, USA

**Keywords:** Genodermatoses, Blaschkolinear, Linear

## Abstract

Linear lichen planus (LP) is a rare dermatologic disease in which lichenoid lesions conform to a blaschkolinear distribution, most commonly on the extremities. Linear discoid lupus erythematosus (DLE) is a cutaneous manifestation of lupus that also conforms to Blaschko’s lines. Blaschkolinear disorders have been shown to result from somatic mosaicism, most recently in nevus sebaceus, epidermal nevi, and syringocystadenoma papilliferum. In linear LP and DLE, presentation of papules along Blaschko’s lines suggests that these disorders can result from keratinocytic genetic mosaicism. Their onset later in life suggests that a secondary trigger is necessary to drive inflammatory reactions to these linear lesions. To date causative mutations have not be identified. We report 2 cases of linear lichen planus and 1 case of linear discoid lupus erythematosus, all histologically confirmed. Both LP patients have experienced episodic regression and recurrence of their lesions in precisely the same distribution, with moderate symptomatic benefit from topical steroids and non-steroidal anti-inflammatories. The DLE patient showed gradual response to hydroxychloroquine over the course of 14 months. These cases highlight linear inflammatory diseases which represent localized variants of disorders for which there are few efficacious therapies. Their linear presentations suggest that they result from somatic mosaicism and genetic investigation of such disorders may reveal relevant therapeutic targets.

## Introduction

Lichen Planus (LP) is a common chronic T-cell mediated inflammatory disorder of unknown etiology [1]. With an estimated worldwide prevalence of between 0.22% and 5% [2], LP can be further subdivided into cutaneous and mucosal subtypes, with the cutaneous form generally appearing as flat, violaceous papules or plaques with a grossly polygonal morphology. Cutaneous LP (CLP) most commonly presents on the flexor surfaces of the extremities, frequently causing mild to moderate pruritus and, rarely, pain. The various presentations of CLP are often grouped into a number of subtypes based on morphological characteristics, distribution, and histopathologic changes. Among these subtypes, linear LP is considered one of the most rare, thought to account for less than 0.2% of all LP presentations [3], and with only 2%–3% of these cases occurring in patients under 20 years of age [4–6]. While linear-appearing LP can manifest at any location secondary to trauma (the Koebner Effect), true linear LP exclusively occurs along developmental lines of Blaschko. Similarly, linear DLE involves the presence of discoid skin lesions, frequently with telangiectasias and hyperpigmentation, localized along lines of Blaschko and without systemic lupus symptoms [7]. The linear variant of DLE is highly rare compared to the standard form, with only approximately 20 case reports in the literature. The Blaschkolinear pattern of this family of diseases likely suggests a keratinocytic determinant to the distribution, with postzygotic mutations in a specific keratinocytic clone following development thought to predispose patients to development of the these disorders [3]. Loss of tolerance to this clone induced by an unknown environmental stimulus then triggers manifestation and symptoms. This form of cutaneous mosaicism has previously been introduced as an explanation of other linear inflammatory disorders, including atopic dermatitis, psoriasis, erythema multiforme, morphea, and vitiligo [8–11], though the specific causative mutations have yet to be identified.

## Report of Cases

### Case 1

**Figure 1:**
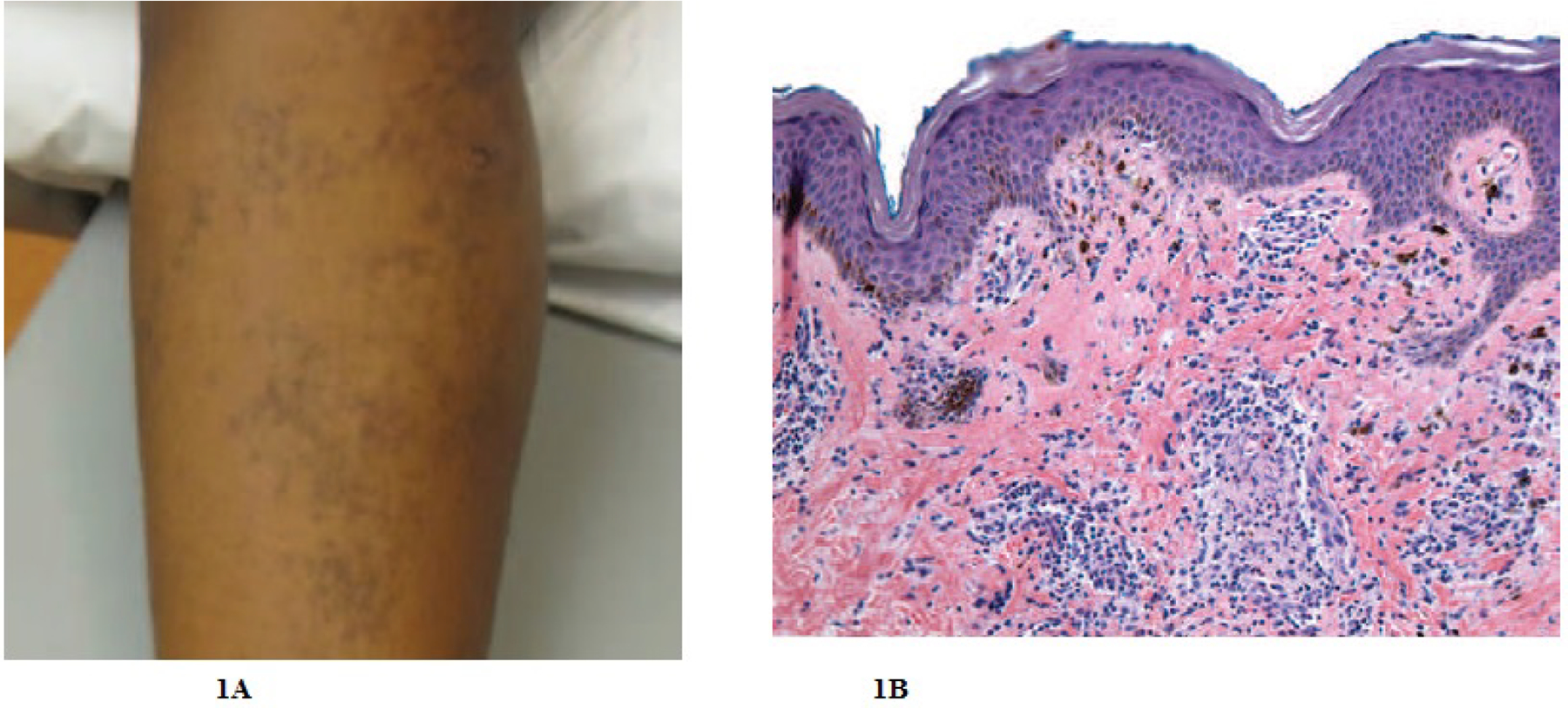
Right Medial Leg Linear Lichen Planus, Case 1: (A) Right medial thigh with approximately one dozen 1–2mm light pink planar papules surrounded by reticulated hyperpigmentation along lines of Blaschko, (B) A biopsy from the right calf showed a patchy lichenoid lymphocytic infiltrate focally obscuring the dermal epidermal junction in association with vacuolar alteration of the basal layer keratinocytes with occasional necrotic keratinocytes and focal overlying parakeratosis. Mild acanthosis, hypergranulosis, and hyperkeratosis noted. Numerous melanophages were seen in the papillary dermis. These findings were consistent with the diagnosis of lichen planus.

A woman in her late teens was referred by her primary physician for a rash on her right leg that first appeared approximately 2 years prior. The eruption initially began as small red and pruritic papules which resolved with significant hyperpigmentation. Her medical history included asthma and atopic dermatitis, but she stated that her leg lesions differed significantly in appearance from her atopic dermatitis. Prior treatments included low potency hydrocortisone cream for symptomatic management. Examination revealed a cluster of approximately one dozen 1–2 mm planar, light-pink papules on the patient’s medial right leg and inner thigh surrounded by reticulated hyperpigmentation extending from the right medial ankle to the right inner thigh in a blaschkoid distribution (Figure 1A). Pathological examination of the lesion was consistent with lichen planus (Figure 1B).

Treatment with medium potency topical steroids was recommended, and follow up showed incremental improvement over the following two months.

### Case 2

**Figure 2:**
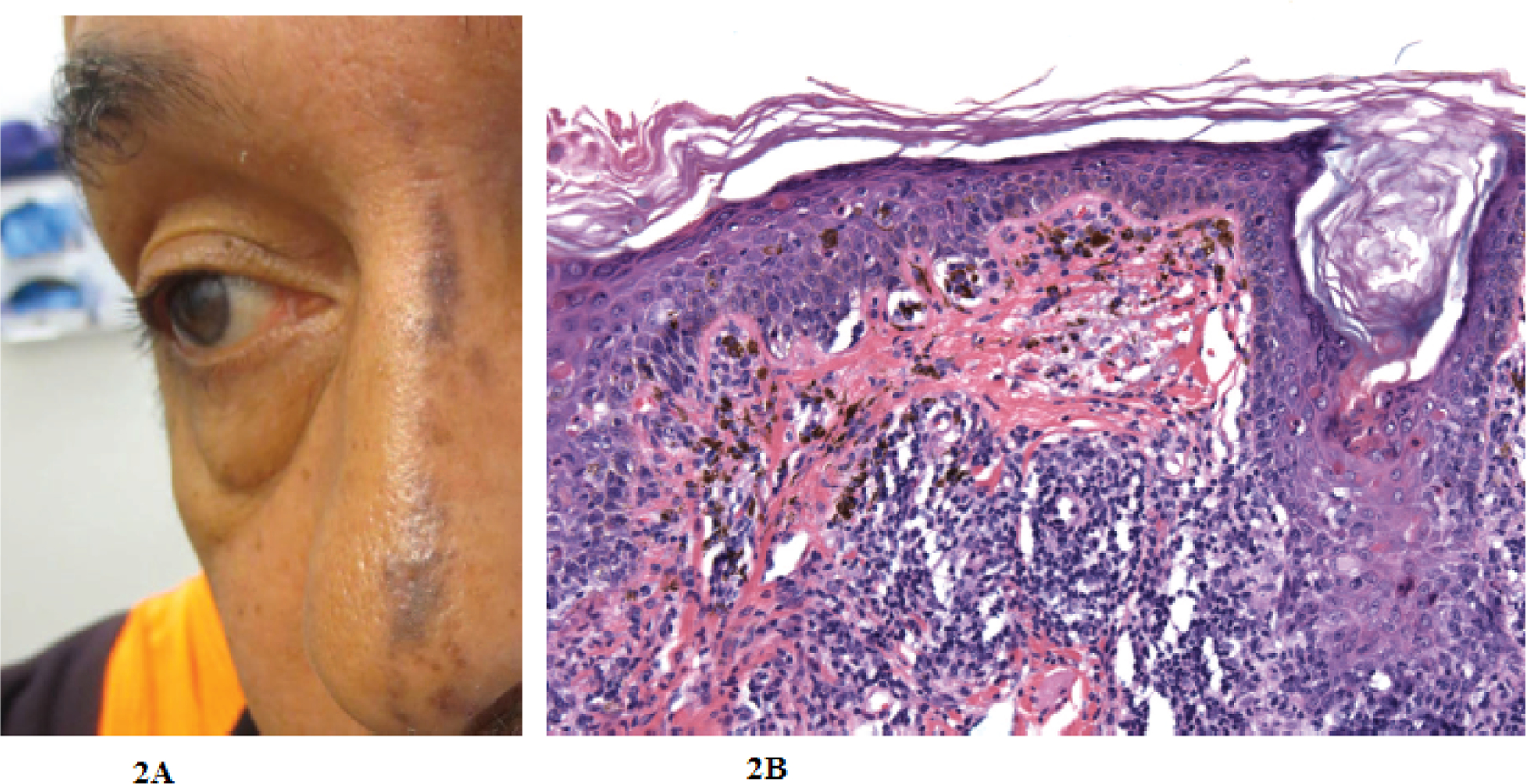
Nasal Linear Lichen Planus, Case 2: (A) Left nasal dorsum with a linear 6 cm × 1 cm linear, thin brown plaque with follicular sparing and perifollicular hyperpigmentation, (B) Skin revealed a patchy interface dermatitis involving the basal aspect of the epidermis and follicular infundibular epithelium. Individual necrotic keratinocytes, some with surrounding lymphocyte “satellitosis,” were noted. Some were superficial to the basal layer, extending occasionally into the stratum granulosum. The infiltrate in the dermis consisted of small lymphocytes with rare plasma cells. Melanophages were conspicuous in the papillary dermis. Hair follicular ostia were patulous and plugged by basket woven orthokeratin.

A man in his early 60’s was referred by his primary physician for an asymptomatic evolving linear rash on his left nasal dorsum which was unresponsive to oral antibiotics given by his primary physician. On exam, a linear 6 cm × 1 cm thin, brown reticulated plaque was present on the nasal dorsum (Figure 2A).

A bisected shave biopsy of skin including epidermis and upper dermis revealed a patchy interface dermatitis involving the basal aspect of the epidermis and follicular infundibular epithelium consistent with lichen planus (Figure 2B). The patient is currently undergoing a trial of topical corticosteroids.

### Case 3

**Figure 3:**
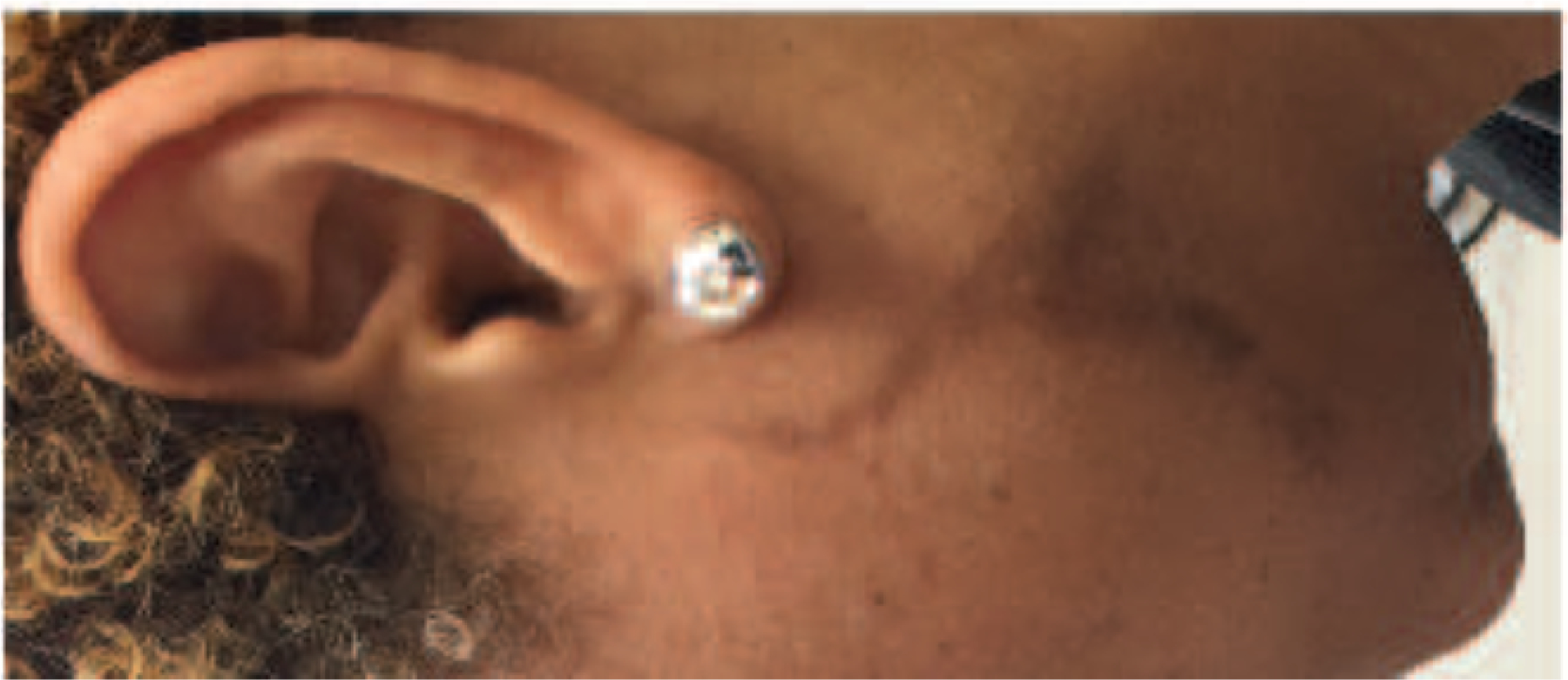
Post-treatment Linear Discoid Lupus, Right Cheek, Case 3 Right cheek with an approximately 5 cm long hyperpigmented patch resulting from resolution of DLE lesion following Blaschko’s lines treated with hydroxychloroquine.

A woman in her mid-40’s presented for a second opinion regarding a new curvilinear, hyperpigmented plaque on her left jawline that had not responded to silvadene cream. The distribution of this plaque coincided with facial lines of Blaschko, and on pathological examination, the histology was consistent with DLE. The patient was treated with hydroxychloroquine 200 mg BID, topical steroids, and daily sunscreen. Within three months, the plaque had clinically resolved, but there was no immediate diminution of hyperpigmentation. Hydroxychloroquine was reduced to 200 mg QOD after 8 months, with further normalization of pigmentation continuing to her last evaluation 14 months after her initial visit (Figure 3).

## Discussion

Here we report two cases of LP and a case of DLE that present along lines of Blaschko. The majority of linear LP lesions are localized to the extremities, with the facial presentation of our second case being highly uncommon [12]. Classic LP occurs in a generalized distribution and is speculated to occur as a response to an as-yet unidentified environmental trigger without obvious delimiting features. In the case of linear LP and DLE, and by extension other linear inflammatory disorders, the strict distribution along lines of embryologic migration suggests that keratinocytes within these regions uniquely permit or stimulate localized inflammation. The clinical differential diagnoses for blaschkolinear inflammatory lesions includes atopic dermatitis, psoriasis, erythema multiforme, morphea, and vitiligo, all of which have variants that occur along ectodermal development lines and may appear similar in the clinical setting. Pathological examination of these lesions should, at the microscopic level, resemble the non-linear forms of these respective diseases, as was the case with the patients described.

Lines of Blaschko represent a Type 1a form of cutaneous mosaicism, which causes keratinocyte precursors of the ectoderm to migrate out from the neural crest along different paths during normal embryological development [13,14]. These lines take the form of V or W-shaped whorls on the trunk and lengthwise-oriented lines along the extremities that are not evident under normal physiological conditions. Pathologies that manifest along these lines, such as lichen striatus and acquired blaschkoid dermatitis, are often readily identifiable in the clinical setting, as they tightly adhere to this distribution pattern, which does not overlap with other important structures such peripheral nerves, or vasculature.

Recent advances in whole exome sequencing have enable genetic investigation of keratinocytic mosaic disorders. Paired whole exome sequencing of peripheral blood and affected tissue have demonstrated mutations in the MAPK pathway causing Nevus Sebaceus (NS) [15], Woolly Hair and Epidermal Nevi (WHEN) [16], and Linear Syringocystadenoma Papilliferum (SCAP) [17]. Future research using similar techniques may ultimately identify the mutations present in blaschkolinear inflammatory disorders that cause or permit disease restricted to individual keratinocyte clones. Improving our understanding of the somatic mutations driving linear inflammatory disorders and how they interact with the immune system represents a novel approach to understanding the root cause of this class of diseases. Once the etiology has been fully characterized, investigators may begin to explore upstream therapeutic targets which may be used to selectively treat and possibly prevent linear inflammatory disorders.

## Conclusion

Linear LP and linear DLE, while rare in and of themselves, represent a family of disorders whose determinants are still poorly characterized. While symptomatic management frequently yields improved quality of life for affected patients, individuals still bear the burden of long-term followup and incomplete palliation of major flares. Strategies for prevention or cure of linear inflammatory lesions remain the ultimate goal of care, with next-generation sequencing representing one potential method to advance our understanding of blaschkolinear disease.
